# Pathways and Associations between Women’s Land Ownership and Child Food and Nutrition Security in Pakistan

**DOI:** 10.3390/ijerph16183360

**Published:** 2019-09-11

**Authors:** Azka Rehman, Qing Ping, Amar Razzaq

**Affiliations:** College of Economics and Management, Huazhong Agriculture University, No.1 Shizishan street, Hongshan District, Wuhan 430070, China; amar.razzaq@hotmail.com

**Keywords:** land right, women’s autonomy, child stunting, malnutrition, gender discrimination, Pakistan

## Abstract

Women’s land ownership plays a noteworthy role in improving various development indicators, including her own wellbeing and children’s food and nutrition security. However, the literature linking women’s access to land rights to the nutritional security of children in Pakistan is limited, even though it is a country facing enormous challenges of childhood malnutrition and gender discrimination. This paper contributes to the existing literature on the benefits of empowering women by studying the association and pathways between women’s land rights and child nutrition, using the 2012–2013 Pakistan Demographic and Health Survey. The ordinary least squares (OLS) regression results indicate that women’s individual land ownership and women’s autonomy in large-scale family purchases have a positive impact on children’s food and nutrition security (FNS). The results of quantile regression (QR) show that these effects are more pronounced in cases of children with severe stunted growth. In addition, a structural equation model shows that the positive relationship between women’s land ownership and child nutrition is partially mediated by women’s increased decision-making power in large-scale household purchases. Our research concludes that ensuring women’s land rights can improve women’s autonomy, which can be an effective policy tool that not only improves women’s welfare but also improves their children’s nutritional security.

## 1. Introduction

Land is not just a physical entity but an important source of livelihood in agriculturally dependent economies. In addition to its financial importance, ownership of land is also viewed as a symbol of power and status [1]. If it is owned by women, the importance of the land will further increase, as the existing literature has widely recognized that when resources are concentrated in women’s hands, it leads to the wellbeing of their residents, more than when resources are controlled by men [2,3]. Following a similar line of argument, previous studies have emphasized that women’s access to land plays a noteworthy role in a range of development issues, including poverty alleviation and food and nutritional security [4]. However, there is a significant gender gap when it comes to land access, because women own only 1–2% of land worldwide [5]. Having no or poor access to land can have serious consequences for women living in countries where the economy depends on agriculture, as they remain dependent on their male relatives for livelihood and meeting the nutritional needs of their children [6].

Among all assets, land is the basic source of women’s survival and development. It gives women stable and secure financial security compared to liquid assets, such as jewelry, flocks and herds [7]. During a financial crisis or family emergency, people are more likely to sell liquid assets that are mainly owned by women, which puts women in an even more vulnerable position [8]. Additionally, land ownership empowers women in two specific ways. (1) It gives her financial security by opening up opportunities for collateral to obtain loans and to expand job opportunities, which in turn increases her financial resources to invest in entrepreneurial activities [9]. (2) A woman’s land ownership also increases her autonomy in the household, which leads to better utilization of income in the form of food and healthcare [9,10]. It has been cited many times that if a woman has higher decision-making power related to household expenditure and her own healthcare, or if she can enjoy higher mobility outside the home, then she can better take care of herself and others in her home [11,12].

Given the significant importance of land in women’s lives, and the surprising presence of a large number of malnourished children, more research is needed to understand the link between women’s land ownership and children’s food and nutritional security. At present, there is a little research on the subject of women’s land ownership in relation to child food and nutritional security. To the best of our knowledge, only two studies have empirically tested the relationship between the aforementioned variables [9,13]. In addition, few studies have investigated the pathways through which women land ownership affects child food and nutritional security [14], although the theoretical foundations of the pathways are well established. Thus, our study will contribute to the existing literature by investigating the pathway and associations between women’s land ownership and children’s food and nutritional status.

Pakistan serves as a suitable case study for this research because it is a country with severe gender discrimination and a poor nutritional status of children. The country ranks second lowest in the global gender index [15] and 38% of children under the age of five have stunted growth. Women’s role in agricultural productivity and household food security in Pakistan is undeniable and significant; 70% of the women labor force is engaged in the agriculture sector [16,17]. However, most of them do not have land ownership and property rights; only 1–2% of women own land autonomously, and about 10% of women share land with their spouses [18], with very few effectively controlling land.

According to the Islamic jurisprudence and the Pakistani constitution, women are entitled to acquire land through gifts, inheritance, will, and purchases. The constitution not only gives women and men equal rights, but also gives the state the right to intervene if someone abolishes the rights of marginalized groups [19]. However, customary practices predominate and deprive women of their land rights. Coupled with a lack of governance, family, social and cultural factors together lead to the denial of women’s land rights.

An exploratory study from Pakistan analyzed the reasons for women’s lack of land access from a detailed field survey and focus group interviews [20]. This study demonstrated that women belonging to higher literacy and economic class were convinced that land ownership can be a source of economic and social status, but that they had to pay a high social price if they claim their rights, in the form of losing respect, relatives, and social legitimacy. The study also pointed out that, due to the country’s dowry system, society considered dowry to be appropriate compensation for the deprivation of women’s land rights, especially when she had no financial responsibility for the family. The exchange marriage and marriage within the family are also common practices in Pakistan. One of the reasons for these marriages is to prevent the transfer of land to other families or to exert social pressure on women, asking them to defer inheritance [21].

All of these factors prevent women from owning land. As a result, land has been passed on to men for generation after generation, leaving women and children vulnerable to poverty and malnutrition. Providing women with land rights is a clear way to address a range of development challenges, including female empowerment and child malnutrition. The current government in Pakistan appears to be committed to ensuring women’s rights to inheritance, as it recently launched an awareness campaign to educate people about the rights of women to inheritance [22]. It remains to be seen whether this move symbolizes the rhetoric or resurgence of women’s inheritance rights in the country. However, the academic literature on this topic is specifically limited, within the context of Pakistan.

The basic purpose of this study is to examine the pathways and associations between women’s land ownership and children’s food and nutrition security, using a range of econometric techniques. We hypothesize that women’s land ownership results in better nutrition security for children in Pakistan. We further hypothesize that the positive impact of women’s land ownership on children’s nutritional security is due to women’s increased autonomy. Therefore, we also test the role of women’s autonomy as a mediator in the proposed relationship between female land ownership and child nutrition. The results of this study are expected to provide useful insights for policymakers in Pakistan.

### 1.2. Literature Review

We briefly review two strands of literature which are pertinent to this study. The first strand of literature supports the argument that land rights help to improve women’s autonomy, while the second strand highlights existing research based on the notion that a woman with higher autonomy benefits her children’s education, health and food, and nutritional security.

#### 1.2.1. Land Ownership and Women’s Autonomy

Women’s autonomy refers to the ability and freedom of women to choose and act independently. It includes the ability of women to make strategic choices, acquire and control resources, and participate in decision-making. Some direct measures of women’s autonomy include women’s access to and control over resources, participation in economic decision-making, self-esteem and mobility [23,24,25]. The land is an important economic resource, especially in agrarian economies, so it may be an indicator of women’s autonomy. Most of the studies found a positive relationship between women’s land ownership and their decision-making power within household. One study from Karnataka, India, found that if a woman owns a house or land, she enjoys higher autonomy related to her own work, healthcare, mobility and household expenditures [26]. In addition, Han et al. [7] empirically demonstrated, using the data of 28 provinces in rural China, that land tenure security promotes women decision-making autonomy in areas such as household purchases, daily necessities purchases, and choice in fertility, medical care, job, and social interaction.

Brule [27] concluded that the amendment in the Hindu Succession Act to give equal rights to sons and daughters in inheritance led to an increase in women’s self-reported decision-making power within the household, and women’s chances to acquire land. They also found that, despite limited substantial impact on equal rights in women’s land share, beneficiary households spent more on women-related goods including health-related expenditure and children’s education. Mishra and Sam [28] investigated the inheritance land reforms in Nepal through empirical testing, and found that women’s land ownership leads to more empowerment in the areas of her decision-making power about her own healthcare, large household purchases and her visits to family and relatives. The authors suggest that, in countries where the economy is largely dependent on agriculture, the provision of land rights to women may lead to higher status and associated welfare effects for women.

The instrumental effect of women’s land ownership on her wellbeing is not restricted only to Asia. Other scholars have also explored these positive outcomes in Latin America and Sub-Saharan Africa. For instance, Wiig [29] empirically estimated the spillover effects of the Peruvian Joint Land Titling Program on female empowerment under the gender equality policy. The author found that women participating in the program were more likely to have higher decision-making power related to expenditure, labor, and investment in comparison to women with no title of the land. Deere and Twyman [10], from research conducted in Ecuador, also reported that if a woman owns a bigger share of household wealth, or if a husband and wife jointly own real estate, then this leads to better decision-making about work and income spending by the couple. Doss et al. [30] found that a woman with autonomous or joint land ownership had higher input in household decision-making processes in three African countries; Mali, Malawi and Tanzania. At the same time, though, they found no similar effects in Orissa, India. Kumar and Quisumbing [31] empirically demonstrated that, in rural Ethiopia, a woman with more surface area of inherited land correlates positively with her overall well-being.

The above discussion shows that there is a positive correlation between women’s land ownership and their decision-making power related to employment, healthcare, mobility, social interaction, investment, family planning, household expenditure, and child education expenditure. The increased autonomy due to land ownership has a positive impact on the overall well-being of women.

#### 1.2.2. Women’s Autonomy and Child Food and Nutritional Security

In the wake of realizing the benefits of female empowerment on development outcomes and human well-being [11], many studies have set out to quantitatively investigate its impacts on a number of children’s food and nutritional indicators. In general, this strand of literature finds that women’s agency has a positive influence on child health outcomes. Three studies from India confirm this finding. First, in rural Andhra Pradesh India, Shroff et al. [32] used longitudinal randomized education intervention and found that children born to women with a higher participation in household decision-making were less likely to be underweight and neglected. Second, another study conducted in India, by using data at three points in time (1993, 1999, 2006), noted that a woman’s free mobility to family and relatives led to better nutritional scores of her child, as compared to women with restricted mobility outside the home [12]. Third, Arulamplam et al. [33], using data from the National Family Health Survey of India, also found that mothers with higher mobility autonomy and decision-making were likely to have a child (under 18 months of age) with less stunted growth.

In addition, Kamiya et al. [34], using the cross-sectional data of 100 mothers and 115 children under the age of five in the Lao PDR found a positive association between childhood development and maternal autonomy, measured by her self-efficacy, self-esteem, and control of money. One study from Pakistan highlighted that women’s decision-making power increases the investment on human development within the household in the form of education and better nutrition [35]. Another study from Pakistan concluded that children living in a household where a woman is the household head (which means she has the autonomy of decision-making), leads to less chances of childhood stunting [36]. In northern Ghana, Tsiboe et al. [37] conclude that women’s empowerment in agriculture across three dimensions, namely production, income control, and leadership, positively influences nutrient availability in the household, while also reducing monetary shortfalls for purchasing food. Cunningham et al. [38] found that, in Nepal, the underlying pathway behind the effect of women’s empowerment in agriculture (five domain index) on children (less than 2 years of age) was seen in the length-for-age z-score (LAZ). They showed that household water, sanitation, and hygiene conditions can mediate the positive relationship between women’s empowerment and children’s LAZ, and suggest that further research is needed to highlight other pathways by which women’s empowerment affects children’s nutritional status.

We have reviewed several studies linking land ownership to women’s autonomy and women’s autonomy to child health outcomes. However, there is little evidence of the direct and indirect impact of women’s land ownership on child food and nutritional security, especially in Pakistan. A recent study in Papua New Guinea found that a father’s assets have a similar effect on child nutritional status as the mother’s assets. The study concluded that the main mechanism of this effect is higher household income rather than women’s bargaining position [14]. However, this study does not consider women’s autonomy as a mediator of the proposed relationship between family assets and child nutrition. Thus, our paper attempts to empirically determine the underlying mechanisms of the relationship between women’s land ownership and child nutritional security.

## 2. Materials and Methods

### 2.1. Data and Variables

The empirical analysis of this study uses the nationally representative Pakistan Demographic and Health Survey (PDHS) data for 2012–2013. This database encompasses information on 3071 children, for whom all socio-economic variables of our econometric specification are available, e.g., education, income, employment, women’s status in the household (decision-making power and land ownership) and anthropometric indicators including stunting and body mass index (BMI). The women in our sample are between the ages of 15 and 49. They have been married at least once in their lives—they could subsequently be divorced or separated, widowed, remarried or could have remained married—and have at least one child under the age of 5. After removing inconsistent and missing information, our sample size was reduced to 2854. We used sample weights to adjust for asymmetric population density in different areas of the country.

The main purpose of this study is to investigate whether women’s land ownership would have a positive effect on child height–age z-score (an anthropometric measure of child food and nutritional security) in Pakistan. Therefore, our primary dependent variable is child growth stunting, which we measured by height-for-age z-score (HAZ score) according to the World Health Organization’s child growth standard. This score compares a child’s z-score to a reference population, and a child is considered to have stunted growth if the HAZ score is less than 2 standard deviation from the reference.

The main independent variable in our study is women’s land ownership. PDHS evaluated women’s land ownership through the question asked of women, “do you own any land either alone or jointly with someone else?” We constructed two variables from this information: one for individual land ownership, and 0 for joint. The variable of women’s individual land ownership takes the value of 1 if a woman owns land alone, otherwise it is 0. Similarly, the variable of woman’s joint land ownership is coded as 1 if a woman owns land jointly with someone else, and it is 0 otherwise. We also analyzed the mechanism behind the relationship of child growth stunting and women’s land ownership. The mediator variable we used is women’s autonomy, which was based on the question “who in the household usually decides on large household purchases”? We coded the response as 1 if a woman participated in the decision-making process and 0 if she had no participation.

In addition to women’s land ownership and autonomy, several other independent variables related to socio-demographic indicators of women and children have been used in the empirical analysis. A list of all the variables used in the analysis and their definitions are provided in Table 1.

### 2.2. Statistical Analysis

Empirically, we applied multiple techniques to find out the relationship between women’s land ownership and child stunting of growth. In addition to this, we also empirically tested the pathways of the aforementioned relationship, which makes our study distinct from the existing literature. First, we used the quantile regression approach in addition to ordinary least squares for point estimation in the conditional distribution of child height-for-age z-score, instead of the mean, by following Kandapal [39] and Imai et al. [12]. Furthermore, we also employed mediation analysis by using structural equation modeling (SEM) to identify the paths.

For econometric analysis, Stata 15 was used with ‘reg’ command along with prefix ‘svy’ for sampling weights to estimate the ordinary least squares model, ‘sqreg’ command for the quantile regression model and ‘gsem’ code for the structural equation model.

#### 2.2.1. Ordinary Least Squares Regression

$$q_{j}=\alpha_{0}+\alpha_{1}AL_{j}+\alpha_{2}JL_{j}+\alpha_{3}X_{j}+e_{j}$$

where, $q_{j}$ is child height-for-age z-score, $AL_{j}$ is women’s land ownership alone while $JL_{j}$ is joint ownership of land with her husband, $X_{j}$, represents all explanatory variables including region, $e_{j}$, is the error term.

#### 2.2.2. Quantile Regression

As explained by Aturupane et al. [40] estimating the mean effects on children’s nutritional score of different socioeconomic variables (e.g., women’s land ownership) can be misleading, because their effect can differ along the conditional nutritional distribution. It is possible that some of the variables may not matter for a child z-score on average, but that it does matter at the lower end of the distribution. Therefore, in addition to simple regression, we also applied the quantile regression model, to better understand the relationship between variables of interest. For policy intervention, it is useful to know that whether women’s land ownership is more valuable for severely stunted children (lower end of distribution) or moderately stunted children (middle of the distribution) or overweight children (higher end of distribution).

Following Koenker and Bassett [41], our equation for ith quantile would take the form:

$$\underset{b\in R^{ℵ}}{\mathrm{Min}}\;\left[\underset{j\in \left\{j:q_{j}\geq X_{t}b\right\}}{\sum }i\left|q_{j}-X_{j}b\right|+\underset{j\in \left\{j:q_{j}<X_{t}b\right\}}{\sum }\left(1-i\right)\left|q_{j}-X_{j}b\right|\right]$$

where, 0<  i<1, $q_{j}$ is child nutritional status (height-for-age z-score) and $X_{j}$ refers to all explanatory variables. Most of the studies used four quartiles (0.1, 0.25, 0.5, and 0.75), but because nutritional cutoff points are more than four, we used 10 quartiles. In our data, there approximately 44% of children are stunted, which is about 50% of the population, so we used five quartiles above and five below the average z-score (0.1, 0.2, 0.3, 0.4, 0.5, 0.6, 0.7, 0.8, and 0.9).

#### 2.2.3. Mediation Analysis

Structural equation modeling (SEM) is a coherent statistical technique used to analyze the mediation effect, with the ability to estimate various multiple regressions or equations altogether. It provides a more effective and direct way to calculate the coefficients of direct and indirect paths and their significance. In this study, we used mediation analysis in the SEM framework to gain information on the underlying mechanism by which women’s land ownership affects child growth stunting. The details on rudimentary channels help project designers and policy thinkers to improve interventions and make cogent policies. Following Cheong and Mackinnon [42], the structural link between the dependent variable, the mediator and the independent variable takes the mathematic form of:

$$\eta_{2}=B\eta_{1}+\Gamma \xi +\zeta$$

where, ‘$\eta_{2}$’ refers to child stunting (endogenous/dependent variable), ‘$\eta_{1}$’ denotes women’s autonomy (endogenous/mediator variable) and ‘ξ’ is to show women’s land ownership (exogenous/independent variable). ‘B’ refers to the direct effect of women’s land ownership on child growth stunting and ‘Γ’ represent the relations of women’s land ownership to women’s autonomy and child growth stunting. ‘ζ’ symbolizes measurement error of the model.

## 3. Results

### 3.1. Descriptive Statistics of Study Population

The basic characteristics of the sample population of children, mothers, and households are shown in Table 2.

#### 3.1.1. Children Characteristics

Table 2 shows the weighted descriptive statistics of the sample population. Child characteristics indicate that the number of boys in the sample (52%) is slightly higher than that of girls. Children of all age groups are evenly distributed across all five age groups, with a slightly lower proportion of children in the 13–25 months age group. The average height-to-age (HAZ) score in the sample was −1.77, indicating poor growth in Pakistani children. The girl’s HAZ score was −1.73, while the boy’s HAZ score was −1.83, indicating that boys’ health is worse than that of the girls.

#### 3.1.2. Mother Characteristics

Mothers’ characteristics show that the average BMI of women in the study population is 23.41, which is within the normal BMI range of 18.5–24.9. However, women in the study population are less educated. The results showed that only 8% of women exceeded secondary education, while 57% were completely illiterate. About 17% of the women in the sample have received primary education. Women’s autonomy-related characteristics indicate that women’s autonomy is low in the study population, because only 28% are employed and only 1% report ownership of land.

#### 3.1.3. Household Characteristics

The household characteristics of the study population indicate that about 70% of mothers and children live in rural areas. In addition, about 44% of children live in poor or poorest families, while 35% of mothers and children come from prosperous families. About 20% of the study population belongs to middle-income families. The average family size of a household is 9. Approximately, more than a quarter of the households do not have a proper toilet facility, however, the majority of households have access to safe drinking water. The regional distribution of sample households represents the national population of these areas.

### 3.2. Multivariate Analysis

#### 3.2.1. Aggregated Analysis

Table 3 and Table 4 report the effects of women’s land ownership (alone and joint) on children’s food and nutritional security. Table 3 presents the estimates for the ordinary least squares model, and Table 4 shows the signs and significance for the estimated coefficients for all variables used in the quantile regression model. All models include a range of socio-economic variables, including child characteristics, maternal attributes, household-level variables, and community characteristics. OLS regression results show that women’s individual land ownership has a significant positive effect on children’s height-for-age z-score (HAZ score). In particular, if a woman owns land, her child’s HAZ score maybe 0.94 points higher than that of women without land. The joint land ownership of women and their husbands has no significant effect on children’s z-scores, although its coefficient has a positive sign. We also analyzed the impact of female land ownership on other important indicators of child nutrition security. Although growth stunting is considered to be one of the most important indicators for measuring a child’s nutritional status, wasting and underweight are also important indicators for measuring short-term lack of nutritional intake. The results show that there is no significant impact of women’s land ownership on wasting as a measure of weight-for-age. However, the impact of women’s individual land ownership on underweight children is positive and significant at 5%, while the impact of joint land ownership is as insignificant as in the case of growth stunting.

Similar results were found from quantile regression. Women’s individual land ownership indicated a positive and significant effect on children’s HAZ score, while women and their husbands’ joint land ownership had no statistically significant effects on the HAZ score of children. In addition, estimates based on quantile regression show that the effect of women’s land ownership is more pronounced at a lower distribution of child height-for-age z-score. The first and third quantiles of the child’s height-for-age z-score are significantly affected by women’s land ownership, while at higher quantiles, women’s land ownership has no significant effects on children’s HAZ score. This suggests that if the child’s growth is more stunted, then the impact of women’s land ownership will be stronger than that of less stunted or normal-growing children.

In addition to women’s land ownership, another relevant variable in the context of female empowerment is women’s autonomy. The existing literature has repeatedly supported the view that that women’s autonomy is the pathway through which women’s land ownership affects child health. Therefore, we added this variable in our models. The estimated coefficients of OLS and QR show that women’s autonomy in large-scale household purchase decisions has a positive effect on reducing child growth stunting. According to OLS estimates, women’s autonomy may increase a child’s HAZ score by 0.17 points compared to non-autonomous women. Furthermore, the point estimation results from QR indicate that the effect of women’s autonomy on reducing stunting in children is stronger when the z-score distribution is lower. These results suggest that women’s autonomy and land ownership may be related to determining child growth stunting. However, we cannot fully understand this mediation through the multivariate analysis presented here. Therefore, in order to further confirm the exact mediation effect of female autonomy, we estimate a structural equation model (Section 3.3).

#### 3.2.2. Disaggregated Analysis

To better understand the link between women’s land ownership and children’s food and nutritional security, we conducted a disaggregated analysis of different regions and urban and rural areas (Table S1 in Supplementary Materials). We found multiple effects. In urban areas, women’s individual and joint land ownership have a positive effect on children’s food and nutrition security, although this effect is statistically significant only for joint land ownership. In rural areas, however, only the effect of individual land ownership is positive and significant. The reason for these results may be that in rural areas, joint ownership leaves women without rights and is mostly dominated by men, which has a negative impact on children’s health. This difference in the use of land rights in a joint ownership scenario leads to serious negative consequences when land is leased to obtain economic benefits. However, in urban areas, land rights are shared by husbands and wives, which allow them to take advantage of co-ownership in the form of more available resources, higher capital investment and risk diversification [9]. These results also indicate that couples may have shared trust and commitment in urban areas, which increases the impact of joint land ownership, and Jackson [43] points out that the lack of such trust and commitment will reduce the successful implementation of joint property rights policies. It can also be implied that in urban areas, customary practices favor women, people are more educated and they are aware of the benefits of empowering women.

After repeating the same analysis at the region level, we found interesting results (Table S2 in Supplementary Materials). The results show that the regions that are more developed and have high literacy rates have a positive coefficient, while in less privileged areas, women’s land ownership does not improve child health. For example, Punjab and Islamabad are the most developed regions of the country, both of which have positive and significant coefficients, supporting the above-mentioned argument.

### 3.3. Mediation Analysis

**Figure 1:**
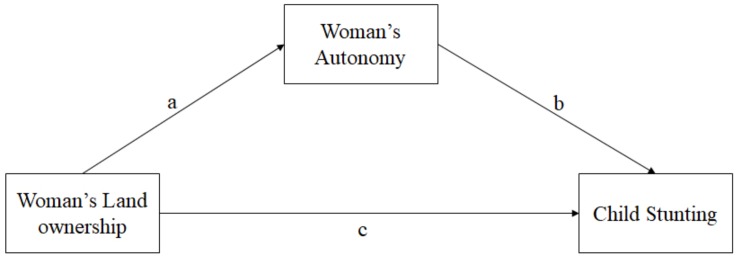
Path diagram of the mediation effect of women’s land ownership on child stunting through women’s autonomy.

Table 5 reports the direct and indirect path coefficients for mediation analysis based on structural equation modeling. The path diagram of the mediation effect of women’s land ownership on child stunting through women’s autonomy is shown in Figure 1. The results show that the influence of women’s land ownership on women’s autonomy in household purchases, as shown by path ‘a’ in Figure 1, is positive and significant. In addition, the estimate of path ‘b’ in Figure 1, indicating the influence of women’s autonomy on child height-for-age z-score, is also positive and significant. These results indicate that women’s land ownership increases women’s autonomy and thus increases the child’s height-for-age z-score.

In the fourth column of Table 5, the indirect effects are given, indicating that women’s autonomy significantly mediates the relationship between her land ownership and child growth stunting. The total effects coefficient, the total effect of women’s land ownership on child growth stunting (the sum of direct and indirect effects), is also positive and significant. Importantly, the total effect found by the mediation analysis is roughly similar to the total effects of the OLS estimates, both of which are significant at the 1% point.

We performed additional analysis following Kassen [14], who argued that women’s land ownership and men’s land ownership have similar effects, and concluded that women’s income generating capacity could mediate this relationship. We repeated the analysis by replacing the land ownership of women with male-owned land, and we found unexpected results. The coefficient of male land ownership is negative for improving child height-for-age z-score, which further supports our hypothesis that women’s autonomy plays a mediating role between women’s land ownership and child nutritional security in the context of Pakistan.

Taken together, these results indicate that women’s land ownership reduces the prevalence of childhood stunting and that women’s autonomy in large-scale family purchases partially mediates this relationship. Women and men sharing land have no significant effect on the child’s height-for-age z-score.

## 4. Discussion

In recent years, the provision of women’s land rights has become a top priority for Pakistan’s development leaders because of their role in improving the household and community welfare. There is ample evidence in developing countries that women’s land ownership can increase women’s autonomy and ultimately affect children’s health outcomes. However, in Pakistan, the literature on women’s land ownership and its impact on women’s autonomy and child health outcomes is very limited. This study not only aims to fill this important gap in the literature, but also strengthens our understanding of the relationship between women’s land ownership and women’s autonomy and its impact on children’s health and nutritional security. By using nationally representative data from Pakistan, this study empirically estimates the links and pathways between women’s land rights and children food and nutritional security. The results indicate that women’s land ownership reduces the incidence of childhood stunting in Pakistan. We also found that women’s decision-making power within the household mediates the association between women’s land ownership and child growth stunting.

The main finding of this study is that women’s land rights help to reduce child growth stunting. This result is consistent with a previous study conducted in Vietnam [9], which also concluded that if a woman has land tenure security, her children are more likely to have better health and education. In the same study, it was found that if a woman owns a family’s land, she can divert the available resources to food and health-related expenditures, away from alcohol and tobacco. In general, our results support efforts to strengthen and promote women’s land rights. However, we did find that the impact of joint ownership between men and women is not significant. Razavi [44] discusses one of the possible reasons for the varying impact of individual and joint land ownership on child stunting, explaining that the benefits of individual or joint land ownership are not upfront. For example, if a woman shares land, she cannot enjoy equal flexibility to manage her plot from production, to claiming land after divorce. Conversely, if a woman owns the land alone, although she has all the rights to use the land, she may face resource constraints, especially if she only owns a small portion of the land.

Another reason for the non-significant impact of joint land ownership may be that the variables used in our study define ownership, but do not clearly indicate who actually controls the land in joint ownership. In developing countries, joint ownership may give men greater power to use the land than women. This means that policies aimed at encouraging men and women to jointly own land should be prudent and can be only implemented after ensuring that women have equal access to land rights. In addition, future research may use more detailed survey data to understand who controls the land in a co-ownership scenario.

Another main finding of this study is that women’s autonomy in durable household purchases mediates the relationship between her land ownership and childhood stunting. It suggests that despite the country’s conservative attitude towards women’s empowerment, women’s land ownership can improve their status in the family and translate into better health outcomes for their children. This finding is away from existing studies who concluded that women’s income generating capacity is the pathway through which women’s land ownership improves child nutritional security [9,14]. One of the reasons for the different results found in our research may be that in Pakistan, due to cultural constraints, few women own land and actually use it to generate income [45]. So land ownership can only help them improve their status in the household. However, our findings are in line with both strands of literature mentioned earlier, that women’s land ownership is positively associated with her decision-making power [46,47,48], and her higher autonomy improves child nutritional status [32,35,36,49].

Based on the above discussion, it is suggested to make women’s land rights strategies more effective through the adoption of an integrated framework approach, while working to change cultural attitudes and institutional arrangements. For example, efforts should be made to promote the mobility of women so that they can truly participate in financial activities and then use these incomes to protect the well-being of themselves and their children. In addition, financial institutions should be designed to enable women to easily access credit [50] and train them to learn the skills needed to manage their land. In fact, despite clear formal laws, customary law predominates in the country, and these laws have little flexibility in empowering women. Therefore, in addition to formulating policies, influential people in society, such as clerics, can also help women and others understand their rights and their benefits to children’s health outcomes.

## 5. Conclusions

This study is especially relevant when taken within the context of the Pakistani government being committed to achieving their Sustainable Development Goals plan in 2030, and that all the authorities have made food and nutritional security a priority challenge. In addition, the current government has recently demonstrated the recognition of women’s inheritance rights and recognizes the importance of women’s empowerment. This study is expected to guide policymakers dealing with FNS issues and provide empirical evidence that while improving women’s lives, their land rights will also lead to better child nutrition security. Our results largely support all efforts or programs designed to encourage women’s land rights. To the best of our knowledge, this study is one of the first studies to directly measure the causal mechanism behind the impact of women’s land ownership on children’s food and nutritional security.

This study concludes that women’s land rights have a positive impact on children’s food and nutritional security, and women’s decision-making power plays a mediating role in this relationship. However, in order to gain potential benefits of women’s land rights, efforts on multiple fronts may be required, including changes in cultural and institutional frameworks in the country. In addition, land rights are particularly crucial for women left alone after divorce, a husband’s migration or death. Thus, independent access to land gives women the opportunity to live a self-reliant life in the wake of unfortunate events, rather than relying on her family or her husband’s family to earn a living.

## Figures and Tables

**Table 1:** Definition of the variables used in this study.

Variables	Definition
Child Characteristics	
Age	Age of the child in months at the time of the survey; grouped into 0–12, 13–24, 25–36, 37–48, 49–60
Gender	Female = 1, Male = 0
Birth order	Birth order of a child in the family
Stunting	Height-for-age z-score
Wasting	Weight-for-age z-score
Underweight	Weight-for-height z-score
Mother characteristics	
Body mass index	Body mass index score calculated by body mass divided by the square of the body height
Education	Education grouped into, no education = 0, primary = 1, secondary = 2, higher than secondary = 3
Age	Age in years at the time of the survey
Employment	Employed = 1, unemployed = 0
Alone land ownership	If a woman owns land alone, yes = 1; no = 0
Joint land ownership	If a woman owns land jointly with her husband, yes = 1; no = 0
Autonomy in large household purchases	If a woman participates in large household purchase (e.g., phone, television, radio, bicycle, car and other durable household belongings) decisions, yes = 1; no = 0
Autonomy in her own healthcare	If a woman participates in decisions about her own healthcare, yes = 1; no = 0
Autonomy in her mobility	If a woman can go outside to meet friends and family without asking someone’s permission. yes = 1; no = 0
Household Characteristics	
Wealth	Wealth index of the household based on number and kind of consumer goods they own; poorest = 1, poorer = 2, middle = 3, richer = 4, richest = 5
Family size	The number of people living in the household
Safe drinking water	If the household has access to safe drinking water, yes = 1, no = 0
Proper toilet facility	If the household has proper toilet facility, yes = 1, no = 0
Place	Living in, urban area = 1, rural = 0
Region/Province	Belongs to, Punjab = 1, Sindh = 2, KPK = 3, Baluchistan = 4, Gilgit = 5, Islamabad = 6

**Table 2:** Descriptive analysis of the sample.

	Mean	SD
Child Characteristics		
Child age (months)	29.653	17.324
Child age between 0–12 months	0.215	0.411
Child age between 13–24 months	0.167	0.373
Child age between 25–36 months	0.209	0.405
Child age between 37–48 months	0.201	0.401
Child age between 49–60 months	0.211	0.408
Female (female = 1)	0.489	0.499
Birth order (range from 1–16)	3.473	2.349
Height-for-age z-score	−1.771	1.713
Height-for-age z-score (Male)	−1.886	1.699
Height-for-age z-score (Female)	−1.655	1.720
**Mother Characteristics**		
Body mass index (range from 13–51)	23.412	5.050
Education (years)	3.382	4.576
No education	0.572	0.495
Primary	0.170	0.376
Secondary	0.176	0.381
Higher than secondary	0.081	0.273
Age (years)	29.194	5.936
Employment (employed = 1)	0.286	0.451
Land ownership (yes = 1)	0.018	0.132
**Household Characteristics**		
Wealth index poorest	0.239	0.427
Wealth index poorer	0.212	0.409
Wealth index middle	0.196	0.397
Wealth index richer	0.196	0.397
Wealth index richest	0.157	0.364
Family size (no.)	9.043	4.758
Safe drinking water (yes = 1)	0.930	0.255
Proper toilet facility (yes = 1)	0.656	0.474
Urban	0.291	0.454
Rural	0.709	0.454
Punjab	0.573	0.495
Sindh	0.229	0.420
KPK	0.138	0.345
Balochistan	0.049	0.216
Gilgit	0.007	0.0849
Islamabad	0.004	0.062

Source: Authors’ calculations based on PDHS 2012–2013.

**Table 3:** Ordinary least squares estimates for child nutritional status.

	Stunting	Wasting	Underweight
Child age			
Child age between 0–12 months	Ref	Ref	Ref
Child age between 13–24 months	−0.79 ***	0.01	−0.19 *
	(0.13)	(0.11)	(0.10)
Child age between 25–36 months	−1.09 ***	0.45 ***	−0.11
	(0.12)	(0.09)	(0.09)
Child age between 37–48 months	−1.03 ***	0.35 ***	−0.22 **
	(0.12)	(0.10)	(0.09)
Child age between 49–60 months	−0.87 ***	0.37 ***	−0.15 *
	(0.12)	(0.10)	(0.09)
Gender (Female = 1, Male = 0)	0.21 ***	0.07	0.13 **
	(0.07)	(0.06)	(0.06)
Child birth order	−0.01	0.02	0.01
	(0.02)	(0.02)	(0.02)
Woman’s ownership of land			
Owns land alone (yes = 1)	0.94 ***	−0.05	0.51 **
	(0.27)	(0.28)	(0.22)
Owns land jointly with her husband (yes = 1)	0.15	−0.21	−0.05
	(0.31)	(0.20)	(0.22)
Woman’s autonomy			
Autonomy in household purchase decision (yes = 1)	0.17*	−0.09	0.04
	(0.10)	(0.07)	(0.08)
Autonomy in her own healthcare (yes = 1)	−0.05	0.12 *	0.06
	(0.10)	(0.07)	(0.08)
Autonomy in her mobility (yes = 1)	0.10	−0.15	−0.03
	(0.14)	(0.11)	(0.10)
Woman’s age	0.02 **	−0.01	0.01
	(0.01)	(0.01)	(0.01)
Woman’s body mass index	0.01	0.03 ***	0.03 ***
	(0.01)	(0.01)	(0.01)
Woman’s education			
No education	Ref	Ref	Ref
Primary	0.12	0.14	0.16 *
	(0.11)	(0.09)	(0.09)
Secondary	0.47 ***	0.13	0.38 ***
	(0.11)	(0.09)	(0.09)
Higher than secondary	0.58 ***	0.32 **	0.58 ***
	(0.15)	(0.13)	(0.12)
Woman’s employment (yes = 1)	0.15 *	0.05	0.11 *
	(0.09)	(0.07)	(0.07)
Family members in the household (no.)	−0.01	0.02 **	0.01
	(0.01)	(0.01)	(0.01)
Wealth index quantiles			
Poorest	Ref	Ref	Ref
Poorer	0.23 *	0.23 **	0.29 ***
	(0.13)	(0.10)	(0.10)
Middle	0.49 ***	0.20 *	0.44 ***
	(0.14)	(0.11)	(0.11)
Richer	0.58 ***	0.15	0.47 ***
	(0.16)	(0.13)	(0.13)
Richest	0.86 ***	0.27 *	0.70 ***
	(0.20)	(0.17)	(0.16)
Place (urban = 1, rural 0)	0.04	0.26 ***	0.21 ***
	(0.10)	(0.08)	(0.08)
Region/Province			
Punjab	Ref	Ref	Ref
Sindh	−0.46 ***	0.05	−0.27 ***
	(0.10)	(0.07)	(0.07)
KPK	0.06	0.23 **	0.18 **
	(0.11)	(0.09)	(0.08)
Balochistan	−1.66 ***	1.28 ***	−0.12
	(0.17)	(0.18)	(0.12)
Gilgit	0.85 ***	1.03 ***	1.19 ***
	(0.22)	(0.17)	(0.16)
Islamabad	0.21 *	−0.18	0.01
	(0.13)	(0.11)	(0.10)
Proper toilet facility (yes = 1)	−0.12	0.13 *	0.00
	(0.10)	(0.08)	(0.07)
Safe drinking water (yes = 1)	−0.09	0.09	0.07
	(0.09)	(0.07)	(0.07)
Constant	−2.36 ***	−2.41 ***	−3.18 ***
	(0.37)	(0.30)	(0.29)
Observations	2854	2854	2854
R-squared	0.19	0.10	0.15

Source: Authors’ calculations using PDHS 2012–2013. Robust standard errors in parentheses *** *p* < 0.01, ** *p* < 0.05, * *p* < 0.1.

**Table 4:** Quantile regression model estimates.

	1st Quantile	2nd Quantile	3rd Quantile	4th Quantile	5th Quantile	6th Quantile	7th Quantile	8th Quantile	9th Quantile
Child age									
Child age between 0–12 months	Ref	Ref	Ref	Ref	Ref	Ref	Ref	Ref	Ref
Child age between 13–24 months	−0.92 ***	−0.83 ***	−0.99 ***	−0.99 ***	−1.12 ***	−1.07 ***	−1.08 ***	−1.22 ***	−1.33 ***
	(0.24)	(0.14)	(0.11)	(0.12)	(0.12)	(0.14)	(0.16)	(0.15)	(0.27)
Child age between 25–36 months	−1.01 ***	−1.16 ***	−1.23 ***	−1.32 ***	−1.45 ***	−1.37***	−1.44 ***	−1.67 ***	−1.71 ***
	(0.17)	(0.15)	(0.08)	(0.10)	(0.11)	(0.13)	(0.12)	(0.16)	(0.26)
Child age between 37–48 months	−0.83 ***	−1.05 ***	−1.23 ***	−1.30 ***	−1.36 ***	−1.37 ***	−1.37 ***	−1.61 ***	−1.98 ***
	(0.17)	(0.11)	(0.11)	(0.14)	(0.12)	(0.10)	(0.13)	(0.15)	(0.24)
Child age between 49–60 months	−0.68 ***	−0.88 ***	−1.02 ***	−1.14 ***	−1.35 ***	−1.42 ***	−1.46 ***	−1.62 ***	−1.83 ***
	(0.16)	(0.16)	(0.11)	(0.13)	(0.13)	(0.16)	(0.15)	(0.18)	(0.26)
Gender (Female = 1, Male = 0)	0.18	0.09	0.08	0.09	0.11	0.09	0.06	0.09	0.12
	(0.11)	(0.07)	(0.07)	(0.06)	(0.08)	(0.07)	(0.07)	(0.09)	(0.14)
Child birth order	−0.04	−0.03	−0.04	−0.04	−0.04*	−0.03	−0.03	−0.00	0.00
	(0.03)	(0.03)	(0.03)	(0.02)	(0.02)	(0.03)	(0.02)	(0.03)	(0.04)
Woman’s ownership of land									
Owns land alone (yes = 1)	0.55 **	0.43	0.46 *	0.32	0.25	0.28	0.29	0.29	0.02
	(0.27)	(0.38)	(0.28)	(0.21)	(0.32)	(0.34)	(0.40)	(0.42)	(0.63)
Owns land jointly with her husband (yes = 1)	0.11	0.08	0.13	−0.02	0.17	0.19	0.26	0.36	0.61
	(0.28)	(0.19)	(0.17)	(0.22)	(0.24)	(0.22)	(0.25)	(0.33)	(0.38)
Woman’s autonomy									
Autonomy in household purchase decision (yes = 1)	0.22	0.29 ***	0.24 ***	0.27 ***	0.18 *	0.19	0.14	0.22 *	0.18
	(0.15)	(0.11)	(0.08)	(0.08)	(0.10)	(0.13)	(0.12)	(0.13)	(0.11)
Autonomy in her own healthcare (yes = 1)	−0.01	−0.14	−0.09	−0.14	−0.02	−0.06	−0.07	−0.02	−0.04
	(0.13)	(0.09)	(0.10)	(0.09)	(0.09)	(0.09)	(0.10)	(0.14)	(0.14)
Autonomy in her mobility (yes = 1)	0.05	−0.11	−0.02	−0.14	−0.12	−0.23	−0.26	−0.09	0.07
	(0.25)	(0.17)	(0.18)	(0.15)	(0.17)	(0.19)	(0.21)	(0.23)	(0.21)
Woman’s age	0.01	0.01	0.01	0.02	0.02 *	0.02 **	0.02 **	0.01	0.02
	(0.01)	(0.01)	(0.01)	(0.01)	(0.01)	(0.01)	(0.01)	(0.02)	(0.01)
Woman’s body mass index	0.01	0.01	0.01	0.02 ***	0.02 ***	0.02 *	0.02 **	0.02 **	0.03 **
	(0.01)	(0.01)	(0.01)	(0.01)	(0.00)	(0.01)	(0.01)	(0.01)	(0.01)
Woman’s education									
Less than primary	Ref	Ref	Ref	Ref	Ref	Ref	Ref	Ref	Ref
Primary	0.15	−0.01	−0.10	−0.00	−0.12	−0.05	0.04	−0.08	0.02
	(0.15)	(0.13)	(0.15)	(0.12)	(0.13)	(0.13)	(0.14)	(0.14)	(0.17)
Secondary	0.42 **	0.24 *	0.15	0.18	0.13	0.20	0.29**	0.17	0.20
	(0.19)	(0.12)	(0.16)	(0.16)	(0.14)	(0.12)	(0.13)	(0.12)	(0.16)
Higher than secondary	0.30	0.41 ***	0.29 *	0.29 *	0.26 *	0.32 *	0.40 **	0.21	0.46 *
	(0.27)	(0.16)	(0.17)	(0.16)	(0.15)	(0.16)	(0.19)	(0.25)	(0.28)
Woman’s employment (yes = 1)	0.27 *	0.22 **	0.11	0.10	0.17	0.19	0.24 *	0.23 **	0.13
	(0.16)	(0.10)	(0.08)	(0.09)	(0.11)	(0.12)	(0.13)	(0.11)	(0.16)
Family members in the household (no.)	−0.01 *	−0.02 ***	−0.03 ***	−0.03 ***	−0.03 ***	−0.02 **	−0.01	−0.02	−0.02
	(0.01)	(0.01)	(0.01)	(0.01)	(0.01)	(0.01)	(0.01)	(0.01)	(0.01)
Wealth index quantiles									
Poorest	Ref	Ref	Ref	Ref	Ref	Ref	Ref	Ref	Ref
Poorer	0.05	0.18	0.11	0.07	0.24 **	0.19	0.18	0.37 *	0.59 **
	(0.23)	(0.15)	(0.13)	(0.11)	(0.12)	(0.17)	(0.17)	(0.19)	(0.29)
Middle	0.21	0.38 ***	0.43 ***	0.45 ***	0.62 ***	0.58 ***	0.60 ***	0.57 ***	0.44 *
	(0.23)	(0.13)	(0.13)	(0.11)	(0.10)	(0.13)	(0.14)	(0.17)	(0.26)
Richer	0.45	0.72 ***	0.70 ***	0.70 ***	0.81 ***	0.72 ***	0.65 ***	0.69 ***	0.68 ***
	(0.28)	(0.14)	(0.14)	(0.12)	(0.13)	(0.13)	(0.15)	(0.17)	(0.23)
Richest	0.68 ***	1.00 ***	1.08 ***	1.05 ***	1.17 ***	1.12 ***	1.00 ***	1.19 ***	1.12 ***
	(0.24)	(0.20)	(0.17)	(0.15)	(0.17)	(0.17)	(0.18)	(0.23)	(0.34)
Place (urban = 1, rural 0)	−0.12	−0.11	−0.05	0.01	0.05	0.08	0.03	0.04	0.15
	(0.13)	(0.11)	(0.09)	(0.09)	(0.11)	(0.09)	(0.11)	(0.14)	(0.16)
Region/Province									
	Ref	Ref	Ref	Ref	Ref	Ref	Ref	Ref	Ref
2.region	−1.02 ***	−0.67 ***	−0.61 ***	−0.50 ***	−0.41 ***	−0.46 ***	−0.42 ***	−0.39 ***	−0.43 **
	(0.16)	(0.13)	(0.12)	(0.09)	(0.09)	(0.09)	(0.08)	(0.11)	(0.17)
3.region	−0.26	−0.10	−0.11	0.01	0.04	−0.02	0.15	0.21	0.39 *
	(0.19)	(0.09)	(0.12)	(0.08)	(0.08)	(0.08)	(0.14)	(0.18)	(0.22)
4.region	−1.80 ***	−1.93 ***	−1.95 ***	−1.89 ***	−1.75 ***	−1.66 ***	−1.62 ***	−1.41 ***	−0.90 ***
	(0.19)	(0.13)	(0.15)	(0.16)	(0.13)	(0.18)	(0.20)	(0.24)	(0.21)
5.region	−0.22	0.12	0.10	0.22	0.48 ***	0.52 **	0.72 **	0.79 ***	1.00 ***
	(0.25)	(0.21)	(0.18)	(0.21)	(0.18)	(0.24)	(0.31)	(0.24)	(0.30)
6.region	0.39	0.36 ***	0.18	0.16	0.21	0.09	0.09	−0.04	0.17
	(0.28)	(0.14)	(0.11)	(0.11)	(0.13)	(0.11)	(0.13)	(0.18)	(0.21)
Proper toilet facility (yes = 1)	−0.08	−0.07	−0.05	0.01	−0.06	−0.04	−0.14	−0.11	0.00
	(0.16)	(0.10)	(0.11)	(0.10)	(0.12)	(0.14)	(0.11)	(0.15)	(0.23)
Safe drinking water (yes = 1)	0.05	0.07	−0.02	−0.07	−0.11	−0.11	−0.16	−0.11	−0.11
	(0.14)	(0.08)	(0.09)	(0.08)	(0.07)	(0.10)	(0.11)	(0.14)	(0.14)
Constant	−3.47 ***	−2.84 ***	−2.29 ***	−2.14 ***	−1.85 ***	−1.68 ***	−1.31 ***	−0.60	−0.48
	(0.61)	(0.50)	(0.42)	(0.35)	(0.42)	(0.42)	(0.34)	(0.47)	(0.58)
									
Observations	2854	2854	2854	2854	2854	2854	2854	2854	2854

Standard errors in parentheses *** *p* < 0.01, ** *p* < 0.05, * *p* < 0.1 Source: Authors’ calculations using PDHS 2012–2013.

**Table 5:** Mediation effect of women’s land ownership on child growth stunting through women’s autonomy.

	**Path Coefficients**		**Indirect Effects**	**Total Effects**
	IV to Mediator (path a)	Mediator to DV (path b)	Point estimates (path ab)	Path (ab + c)
Women’s autonomy in household purchase decision	0.449 ** (0.212)	0.260 *** (0.081)	0.117 * (0.066)	1.197 *** (0.302)

We control both paths with a range of socioeconomic variables including child, woman and household characteristics. DV: Dependent variable, IV: Independent variable. Standard errors in parentheses *** *p* < 0.01, ** *p* < 0.05, * *p* < 0.1 Source: Authors’ calculations based on PDHS 2012–2013.

## Supplementary Materials

The following are available online at https://www.mdpi.com/1660-4601/16/18/3360/s1, Table S1: Ordinary least squares estimates for child nutritional status of urban and rural regions, Table S2: Ordinary least squares estimates for child nutritional status in different regions.
