# Supplementary material for: Pathways and Associations between Women’s Land Ownership and Child Food and Nutrition Security in Pakistan
Source: Int J Environ Res Public Health. 2019 Sep 11;16(18):3360. doi: 10.3390/ijerph16183360 (PMC6765811; doi:10.3390/ijerph16183360)
Supplement: Supplementary file 1 [file ijerph-16-03360-s001.pdf]

Table S1: Ordinary least square estimates for child nutritional status of urban and rural regions

	Urban	Rural
Child age		
Child age between 0-12 months	Ref	Ref
Child age between 13-24 months	-0.65***	-0.85***
	(0.23)	(0.16)
Child age between 25-36 months	-1.05***	-1.12***
	(0.21)	(0.15)
Child age between 37-48 months	-0.91***	-1.07***
	(0.19)	(0.16)
Child age between 49-60 months	-0.86***	-0.89***
	(0.19)	(0.15)
Gender (Female=1, Male=0)	0.19	0.23**
	(0.12)	(0.09)
Child Birth order	-0.03	-0.01
	(0.04)	(0.03)
Woman's ownership of land		
Own land alone (yes=1)	0.71	1.02***
	(0.66)	(0.31)
Owns land jointly with her husband (yes=1)	1.17***	-0.41
	(0.40)	(0.36)
Woman's autonomy		
Autonomy in household purchasing decision (yes=1)	0.23	0.15
	(0.16)	(0.13)
Autonomy in her own health care (yes=1)	-0.05	-0.03
	(0.16)	(0.13)
Autonomy in her mobility (yes=1)	0.23	0.03
	(0.26)	(0.16)
Woman's age	0.01	0.02**
	(0.01)	(0.01)
Woman's body mass index	0.01	0.02**
	(0.01)	(0.01)
Woman's education		
No education	Ref	Ref
Primary	0.24	0.05
	(0.18)	(0.14)
Secondary	0.63***	0.39***
	(0.18)	(0.15)
Higher than secondary	0.69***	0.60***
	(0.21)	(0.22)
Woman's employment (yes=1)	0.11	0.17
	(0.14)	(0.11)
Family members in the household (no.)	-0.02	-0.00
	(0.01)	(0.01)
Wealth index quintiles		
Poorest	Ref	Ref

Poorer	0.57	0.27*
	(0.41)	(0.14)
Middle	0.96***	0.50***
	(0.36)	(0.16)
Richer	0.68*	0.77***
	(0.36)	(0.20)
Richest	1.08***	0.67**
	(0.38)	(0.29)
Region/Province		
Punjab	Ref	Ref
Sindh	-0.64***	-0.36**
	(0.13)	(0.14)
KPK	0.04	0.15
	(0.16)	(0.13)
Balochistan	-1.19***	-1.73***
	(0.21)	(0.22)
Gilgit	-0.19	1.19***
	(0.25)	(0.27)
Islamabad	0.38**	0.19
	(0.18)	(0.19)
Proper toilet facility (yes=1)	0.29	-0.19*
	(0.22)	(0.12)
Safe drinking water (yes=1)	-0.21	0.08
	(0.14)	(0.13)
Constant	-2.23***	-2.77***
	(0.51)	(0.37)
Observations	1,223	1,631
R-squared	0.21	0.19

Source: Authors' calculations using PDHS 2012-13

Robust standard errors in parentheses

*** p<0.01, ** p<0.05, * p<0.1

Table S2: Ordinary least square estimates for child nutritional status in different regions

	Punjab	Sindh	KPK	Baluchistan	Gilgit	Islamabad
Child age						
Child age between 0-12 months	Ref	Ref	Ref	Ref	Ref	Ref
Child age between 13-24 months	-0.76***	-0.79***	-1.06***	-1.11*	-1.75***	-1.52***
	(0.18)	(0.23)	(0.33)	(0.60)	(0.62)	(0.40)
Child age between 25-36 months	-1.11***	-0.84***	-1.22***	-2.02***	-2.31***	-1.72***
	(0.16)	(0.24)	(0.31)	(0.49)	(0.50)	(0.38)
Child age between 37-48 months	-0.92***	-1.25***	-1.01***	-1.30***	-2.73***	-1.71***
	(0.17)	(0.22)	(0.33)	(0.50)	(0.45)	(0.34)
Child age between 49-60 months	-0.74***	-0.94***	-0.99***	-2.20***	-2.51***	-1.62***
	(0.17)	(0.21)	(0.33)	(0.52)	(0.46)	(0.37)
Gender (Female=1, Male=0)	0.18*	0.31**	0.22	0.25	0.04	-0.15
	(0.10)	(0.14)	(0.19)	(0.28)	(0.31)	(0.20)
Child Birth order	-0.00	-0.00	0.02	-0.26**	-0.04	0.02
	(0.03)	(0.05)	(0.06)	(0.10)	(0.10)	(0.08)
Woman's ownership of land						
Own land alone (yes=1)	1.20***	-1.49***	-0.35	-0.34	-1.91	0.68
	(0.28)	(0.48)	(0.55)	(0.64)	(1.43)	(0.60)
Own land jointly with her husband (yes=1)	0.61	1.05	-0.67	1.18	0.22	1.08***
	(0.63)	(1.13)	(0.44)	(0.87)	(0.34)	(0.41)
Woman's autonomy						
Autonomy in household purchase decision (yes=1)	0.15	0.48**	-0.04	0.59	-0.33	-0.16
	(0.14)	(0.22)	(0.28)	(0.56)	(0.45)	(0.29)
Autonomy in her own health care (yes=1)	-0.05	0.08	-0.17	-0.37	-0.32	0.21
	(0.13)	(0.21)	(0.27)	(0.53)	(0.36)	(0.26)
Autonomy in her mobility (yes=1)	0.37*	-0.27	0.28	-0.39	-0.97**	-0.34
	(0.19)	(0.22)	(0.37)	(0.54)	(0.42)	(0.28)
Woman's age	0.01	0.04*	0.03	0.11**	-0.00	-0.04*
	(0.01)	(0.02)	(0.02)	(0.04)	(0.03)	(0.02)
Woman's body mass index	0.01	0.01	0.03	-0.02	-0.00	0.03
	(0.01)	(0.02)	(0.02)	(0.04)	(0.04)	(0.02)
Woman's education						
No education	Ref	Ref	Ref	Ref	Ref	Ref

Primary	0.20	0.04	-0.29	-0.10	-0.64	-0.79**
	(0.15)	(0.19)	(0.30)	(0.58)	(0.40)	(0.33)
Secondary	0.46***	0.75***	0.27	-0.26	-0.00	-0.84**
	(0.15)	(0.27)	(0.25)	(0.70)	(0.41)	(0.35)
Higher than secondary	0.69***	0.65*	0.57	-1.67**	-0.80	-0.62*
	(0.20)	(0.35)	(0.35)	(0.81)	(0.52)	(0.36)
Woman’s employment (yes=1)	0.07	0.33**	0.36	-0.12	3.63***	0.26
	(0.11)	(0.16)	(0.36)	(0.35)	(1.07)	(0.29)
Family members in the household (no.)	-0.01	-0.01	-0.01	-0.03	-0.06**	-0.02
	(0.01)	(0.02)	(0.02)	(0.03)	(0.03)	(0.03)
Wealth index quintiles						
Poorest	Ref	Ref	Ref	Ref	Ref	Ref
Poorer	0.31	0.17	0.36	-0.19	0.17	1.60**
	(0.21)	(0.23)	(0.39)	(0.39)	(0.37)	(0.81)
Middle	0.68***	-0.05	0.47	0.59	-0.17	1.42***
	(0.20)	(0.33)	(0.41)	(0.58)	(0.48)	(0.40)
Richer	0.79***	0.03	0.49	1.33**	0.93	1.41***
	(0.23)	(0.37)	(0.44)	(0.67)	(0.60)	(0.41)
Richest	1.02***	0.20	1.06**	1.32*	-0.66	2.13***
	(0.28)	(0.42)	(0.51)	(0.75)	(1.45)	(0.40)
Place (urban=1, rural 0)	0.03	0.01	0.15	-0.64*	0.36	-0.25
	(0.13)	(0.24)	(0.21)	(0.37)	(0.33)	(0.20)
Proper toilet facility (yes=1)	-0.16	0.01	-0.11	-0.33	-0.12	0.08
	(0.14)	(0.27)	(0.27)	(0.35)	(0.44)	(0.35)
Safe drinking water (yes=1)	-0.02	0.21	0.07	-0.58	-1.10***	0.23
	(0.12)	(0.22)	(0.21)	(0.47)	(0.38)	(0.18)
Constant	-2.17***	-3.44***	-2.96***	-2.31	1.95	0.03
	(0.46)	(0.76)	(0.88)	(1.69)	(1.44)	(0.82)
Observations	889	667	517	285	291	205
R-squared	0.19	0.15	0.10	0.28	0.33	0.34

Source: Authors' calculations using PDHS 2012-13

Robust standard errors in parentheses

*** p<0.01, ** p<0.05, * p<0.1
